# Physiological Stress and Refuge Behavior by African Elephants

**DOI:** 10.1371/journal.pone.0031818

**Published:** 2012-02-22

**Authors:** David S. Jachowski, Rob Slotow, Joshua J. Millspaugh

**Affiliations:** 1 Department of Fisheries and Wildlife Sciences, University of Missouri, Columbia, Missouri, United States of America; 2 Amarula Elephant Research Programme, School of Life Sciences, University of KwaZulu-Natal, Durban, South Africa; University of Western Ontario, Canada

## Abstract

Physiological stress responses allow individuals to adapt to changes in their status or surroundings, but chronic exposure to stressors could have detrimental effects. Increased stress hormone secretion leads to short-term escape behavior; however, no studies have assessed the potential of longer-term escape behavior, when individuals are in a chronic physiological state. Such refuge behavior is likely to take two forms, where an individual or population restricts its space use patterns spatially (spatial refuge hypothesis), or alters its use of space temporally (temporal refuge hypothesis). We tested the spatial and temporal refuge hypotheses by comparing space use patterns among three African elephant populations maintaining different fecal glucocorticoid metabolite (FGM) concentrations. In support of the spatial refuge hypothesis, the elephant population that maintained elevated FGM concentrations (iSimangaliso) used 20% less of its reserve than did an elephant population with lower FGM concentrations (Pilanesberg) in a reserve of similar size, and 43% less than elephants in the smaller Phinda reserve. We found mixed support for the temporal refuge hypothesis; home range sizes in the iSimangaliso population did not differ by day compared to nighttime, but elephants used areas within their home ranges differently between day and night. Elephants in all three reserves generally selected forest and woodland habitats over grasslands, but elephants in iSimangaliso selected exotic forest plantations over native habitat types. Our findings suggest that chronic stress is associated with restricted space use and altered habitat preferences that resemble a facultative refuge behavioral response. Elephants can maintain elevated FGM levels for ≥6 years following translocation, during which they exhibit refuge behavior that is likely a result of human disturbance and habitat conditions. Wildlife managers planning to translocate animals, or to initiate other management activities that could result in chronic stress responses, should consider the potential for, and consequences of, refuge behavior.

## Introduction

In responding to real or perceived threats, vertebrates initiate a physiological stress response that has broad implications if stress levels are maintained at a high level (i.e., chronic) [1]. The production of stress hormones is a key physiological step in balancing the expenditure of energy, and facilitates the ability of an individual to survive exposure to a stressor [2], [3]. While this response is effective in the presence of short-term stressors, chronic levels of stress can result in various pathological dysfunctions, including an increase in blood glucose, or the inhibition of reproduction, immune function, or growth [1], [4]. Therefore, while short-term releases of stress hormones help a vertebrate adapt to its surroundings, over extended periods of time, chronic release of hormones should be minimized to reduce deleterious effects [2].

Vertebrates limit chronic exposure to stressors through three kinds of facultative behavioral responses [5]: (1) the individual exhibits escape behavior away from the perturbation; (2) the individual remains in the area, but identifies and uses a refuge to avoid the perturbation; and (3) the individual identifies and uses a refuge, but will move outside the refuge during periods of non-disturbance. Many studies have focused on short-term escape behavior away from disturbances [5], [6]. The latter two kinds of responses have received considerably less attention. Previous studies have suggested that use of refugia typically is temporary, and that normal space use continues once the disturbance passes [5], [7]. However, to our knowledge, there has been no research to evaluate if long-term use of refugia is likely to occur if the animal does not adjust to the source of perturbation, and maintains a chronic physiological state.

Descriptions of wildlife use of “refuges” or “refugia” are increasingly widespread in ecology and conservation biology. In the ecological literature, refugia frequently are defined by fine-scale spatial responses of animals to perturbations [8], [9], [10]. While particular behaviors and space use patterns have been reported as refuge behavior, little is known about the facultative process behind those observations. Initiation of refuge behavior is an active process involving an external cue (i.e. the stressor), internal physiological response, and active movement and selection of refugia [11]. The extent to which physiological state influences the timing and duration of refuge behavior is poorly understood, despite its potential importance in predicting when, where, to what extent, and for how long refuge behavior will occur.

The refuge behavior of African elephants (*Loxodonta africana*) is relatively well documented through long-term behavioral studies. Elephants are long-lived with high cognitive ability for spatial memory [12] that allows them to adapt space use patterns based on the location of resources [13], boundaries [14], or past experiences [15]. Behavioral observations suggest that elephants exhibit at least two facultative behavioral responses indicative of spatial and temporal refuge behavior. Firstly, humans have restricted elephant movements, and fragmented habitat, through the creation of real (e.g., electric fences) or perceived (e.g., human land use and disturbance) boundaries [16]. In response, elephants have restricted space use patterns and have identified, used, and rarely occurred outside of protected areas or refugia [17]. Secondly, in addition to restricting movements spatially, space use can be modified temporally to avoid areas during periods of disturbance [18], [19]. This pattern of spatio-temporal refuge behavior allows elephants to reoccupy habitats when humans are absent [20], [21].

In South Africa, where elephants are being reintroduced to relatively small fenced reserves, there is a particular need to consider the potential for refuge behavior. Elephants have been translocated for reintroduction into over 58 reserves in South Africa [22]. The process of translocation is well established and designed to be as unobtrusive to the animals as possible [23], but still results in an elevated physiological stress response for up to 30 days post-release [24], [25]. However, little is known about the potential for longer-term stress responses in elephants following translocation [26], despite the need to understand how they habituate to their new surroundings, and if they exhibit aberrant behavior that poses a risk to elephants, other animals and people [23]. To facilitate acclimatization, it has been suggested that managers provide “refuge areas” to allow translocated elephants freedom from harassment [27]. Thus, there is interest in identifying when and where refuge behavior occurs, to mitigate potential human-elephant conflict.

In this study, we evaluated spatial and temporal hypotheses of refuge behavior in elephants by comparing space use patterns among three restored elephant populations. These populations maintained different levels of physiological stress, including one with chronic levels. Under the spatial refuge hypothesis, where individuals restrict space use when stress hormone levels are elevated, we expected elephant populations that were chronically stressed to avoid disturbance by exhibiting restricted space use patterns. Therefore, we examined two metrics: home range size, and the proportion of the area used by elephant family groups in each reserve. Under the temporal refuge hypothesis, where individuals temporally alter their use of space when stress hormone levels are elevated, we expected elephant family groups in a state of chronic stress to restrict their use of space during the day, when human disturbance existed, and increase their use of space at night, when disturbance ceased. We tested support for the temporal refuge hypothesis by evaluating whether elephant family group home range sizes were smaller during the day than at night, whether family groups used the same areas during the day and night, and whether seasonal resource selection differed between night and day. By comparing these metrics across elephant populations in different physiological states, we were able to link stress with refuge behavior.

## Results

**Figure 1:**
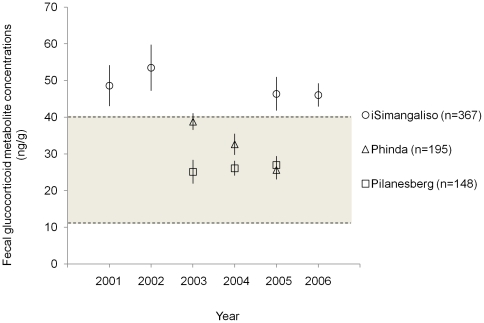
Fecal glucocorticoid metabolite values of elephants in each reserve. Average (with 95% confidence intervals) fecal glucocorticoid metabolite (FGM) concentrations (in dry weight ng/g) for each year samples were collected. Basal FGM concentrations for elephants (15–40 ng/g) are shaded grey.

From 2000 to 2006, we collected and assayed 709 fecal samples from elephant populations in the three reserves included in this study (Phinda Private Game Reserve n = 195; iSimangaliso Wetland Park n = 366; Pilanesberg National Park n = 148). Fecal glucocorticoid metabolite concentrations were significantly higher for elephants in iSimangaliso than for elephants in the other two reserves (*F*_2, 708_ = 80.17, *P*<0.0001) (Figure 1). Elephants in iSimangaliso consistently had FGM concentrations around 50 ng/g, indicative of a chronic stress response [25], [28]. In comparison, elephants in Phinda and Pilanesberg had relatively moderate FGM concentrations (25–35 ng/g), typical of baseline levels in elephants [25], [28] (Figure 1). Across all reserves, FGM values were 20% higher in the dry season than in the wet season (*F*_1, 705_ = 23.20, *P*<0.0001). We observed differences in FGM levels among years (*F*_5, 700_ = 2.79, *P* = 0.0167). However, annual differences primarily occurred in FGM concentrations of elephants in Phinda; FGM levels of elephants in iSimangaliso were consistently elevated across all years (Figure 1).

**Figure 2:**
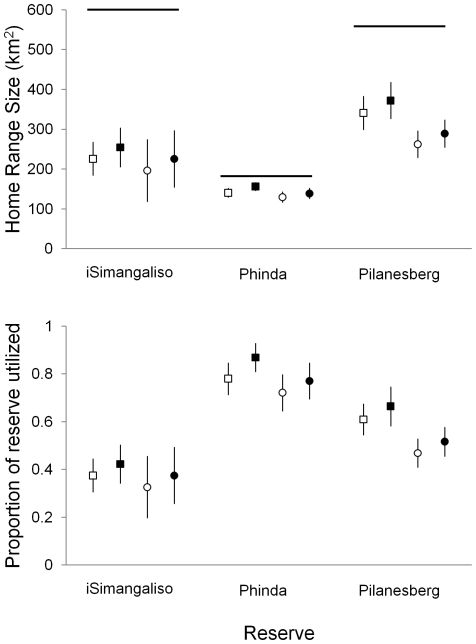
Home range size of elephants in each reserve. Average (with 95% confidence interval) home range size (km^2^) during the wet (squares) and dry (circles) seasons (top graph). Horizontal lines indicate the size of each reserve. The bottom graph depicts the average (with 95% confidence interval) proportion of each reserve occupied by elephant home ranges. Solid symbols represent mean average home range sized based on utilization distributions (UDs) calculated from nighttime locations and hollow symbols represent UDs calculated from daytime locations.

**Figure 3:**
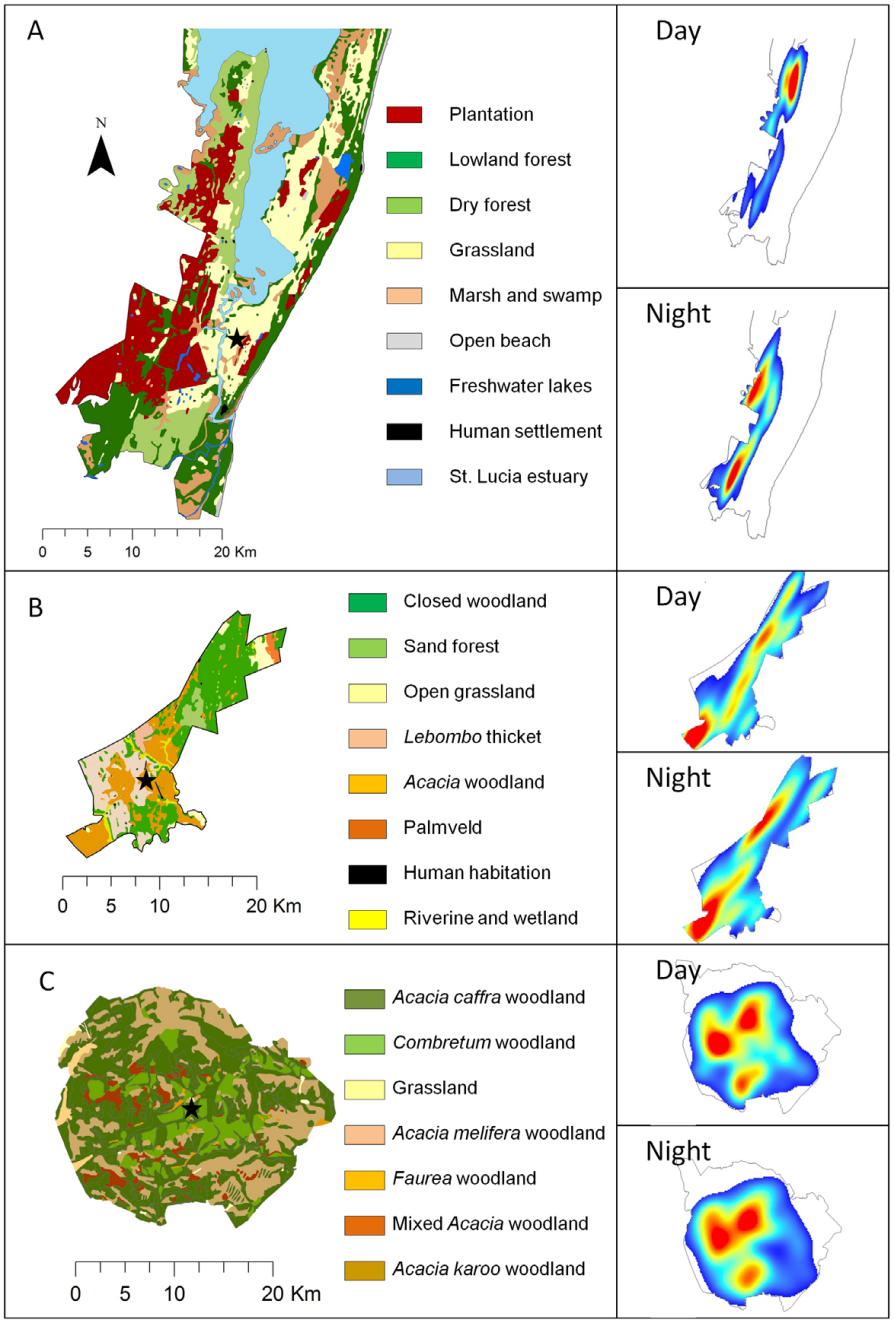
Space use patterns by elephants in each reserve. The distribution of habitat types within iSimangaliso Wetland Park (A), Phinda Private Game Reserve (B), and Pilanesberg National Park (C). The star within each reserve represents the location of the boma (or preconditioning enclosure) that was also the initial release site of elephants. Inset on the right are 95%fixed kernel seasonal utilization distributions (UDs) for a select adult female elephant in each of our study areas based on daytime (top) and nighttime (bottom) locations. Areas in red within the UD represent areas of high intensity use, which fade to blue in areas of low use, and reserve boundaries are demarcated by solid lines. Space use was restricted and differed between day and night at iSimangaliso Wetland Park (A), compared to Phinda Private Game Reserve (B) and Pilanesberg National Park (C).

In support of the spatial refuge hypothesis, from 2004 to 2007 elephants in iSimangaliso maintained smaller home ranges and used a smaller proportion of the reserve compared to elephants in the other two populations. Despite iSimangaliso being slightly larger (602 km^2^) than Pilanesberg (560 km^2^) (Figure 2), elephant home range size was twice as large in Pilanesberg than in iSimangaliso (*F*_2, 52_ = 48.45, *P*<0.0001). Within all reserves, home range size was consistent across years (*F*_4, 52_ = 1.66, *P* = 0.1744), but on average 65 km^2^ larger during the wet as opposed to the dry season (*F*_1, 52_ = 18.47, *P*<0.0001). When scaled in proportion to the total area available within the reserve, elephant home ranges in iSimangaliso occupied 20% less of the available area (*x* ¯ = 0.35, SE = 0.04, range = 0.13–0.56), than in the similarly-sized Pilanesberg (*x* ¯ = 0.55, SE = 0.03, range = 0.17–0.74), and 43% less than in the smaller Phinda (180 km^2^) (*x* ¯ = 0.78, SE = 0.02, range = 0.63–0.98) (Figure 2). Elephants utilized more of the available area during each season in the relatively small Phinda reserve than in the other two reserves (*F*_2, 52_ = 49.29, *P*<0.0001) (Figure 3). Similar to home range size, scaled home ranges were consistent across years (*F*_4, 52_ = 2.02, *P* = 0.1059), but on average elephants utilized 9% more of the reserve during the wet as opposed to the dry season (*F*_1, 52_ = 21.14, *P*<0.0001).

**Figure 4:**
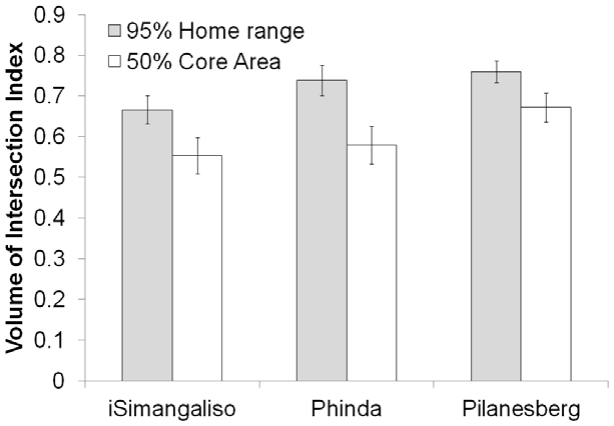
Day vs. night space use overlap by elephants in each reserve. Mean (with 95% confidence interval) volume of intersection index scores for elephant based on comparisons between day and night home range (grey) and core area (white) space use patterns. Volume of intersection index statistic measures the amount of overlap between two utilization distributions. Index values range from 0 to 1, where higher scores indicate a higher degree of overlap.

We found mixed support for our temporal refuge hypothesis. We found no difference between day and night home range sizes of elephants within any reserve (iSimangaliso, *F*_6, 16_ = 0.20, *P* = 0.9706; Pilanesberg, *F*_12, 48_ = 0.27, *P* = 0.9921; Phinda, *F*_10, 18_ = 0.39, *P* = 0.9324) (Figure 2). However, across reserves, we observed significantly less day vs. night space use overlap in iSimangaliso compared to Pilanesberg and Phinda (Figures 3 and 4), at both the home range (*F*_2, 47_ = 7.52, *P* = 0.0015) and core area (*F*_2, 46_ = 8.26, *P* = 0.0009) scales. In iSimangaliso, we observed 66.6% overlap in daytime and nighttime space use at the home range scale and 55% space use overlap at the core area scale (Figure 4). By contrast, we observed 7–10% more overlap in daytime and nighttime space use in Pilanesberg and Phinda at the home range scale, and 8–10% more at the core area scale. The amount of day-night space use overlap did not differ by season (home range, *F*_1, 47_ = 0.42, *P* = 0.5225; core area, *F*_1, 46_ = 0.04, *P* = 0.8346) or year of investigation (home range, *F*_1, 47_ = 1.74, *P* = 0.1573; core area, *F*_1, 46_ = 1.32, *P* = 0.2751).

**Figure 5:**
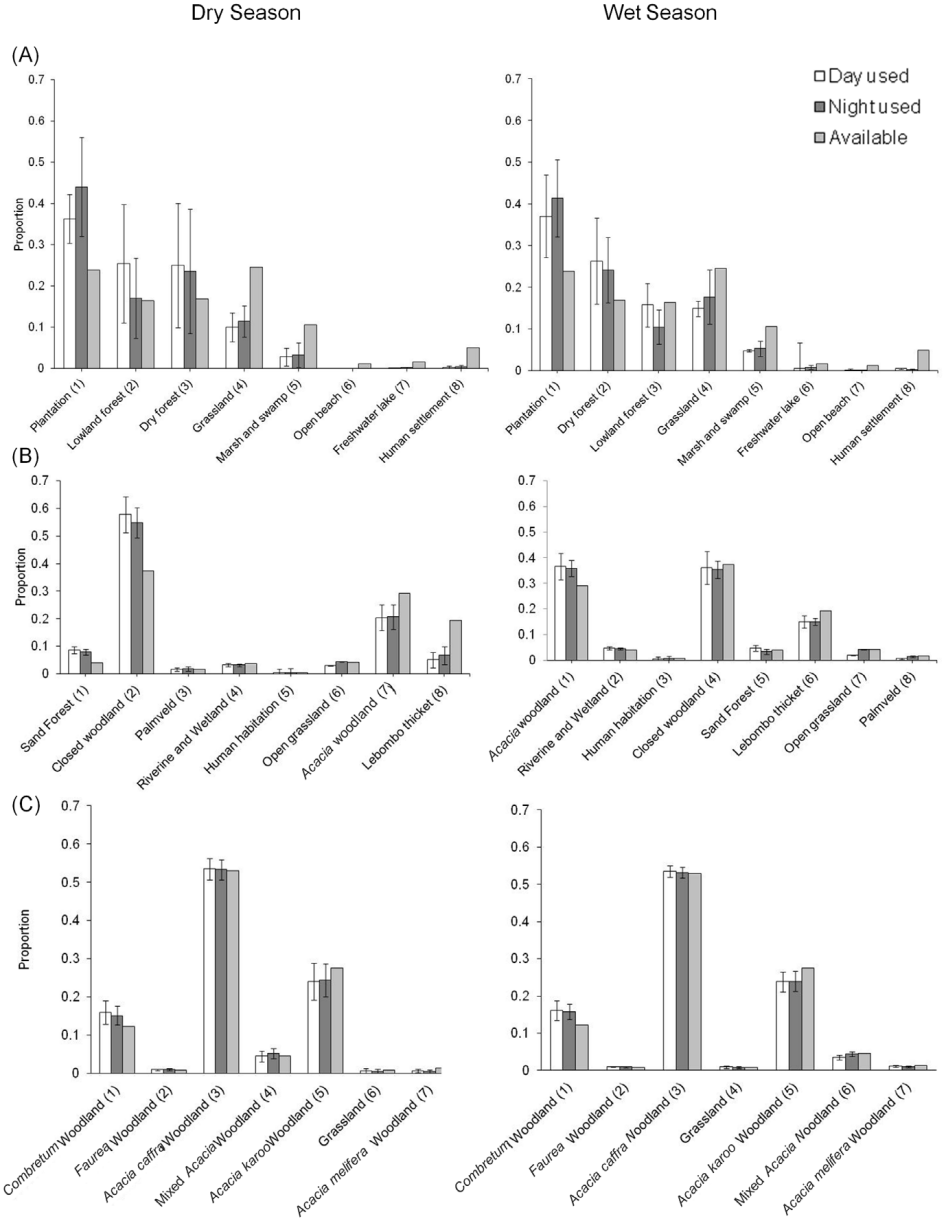
Compositional analysis of habitat use by elephants between day and night among reserves. Mean (with 95% confidence intervals) weighted day and night time use (calculated by summing UD fixed kernel scores by habitat type), compared to availability of habitat types at the reserve level. Habitat names are followed by their compositional analysis rank (Aebischer et al. 1993). Graphs are separated by dry (left column) and wet (right column) season as well as by reserve in rows: iSimangaliso Wetland Park (A), Phinda Private Game Reserve (B), and Pilanesberg National Park (C).

In terms of resource selection patterns, in iSimangaliso, with the exception of dry forest, elephants selected forest plantation over all other habitat types (Table S1). This pattern was consistent across seasons (Pillai's Trace = 1.3258, *F*_21, 30_ = 1.13, *P* = 0.3713) and time of day (Pillai's Trace = 1.4295, *F*_42, 78_ = 0.58, *P* = 0.9718), suggesting that elephants generally tended to select forest plantation in favor of most native habitat types regardless of time of day or their relative availability (Figure 5). In contrast, in Phinda and Pilanesberg where tree plantations were not present, elephants exhibited differing seasonal resource selection patterns that favored native forest habitats (Table S1). In Phinda, we observed seasonal differences in resource selection (Pillai's Trace = 2.0965, *F*_35, 80_ = 1.65, *P* = 0.0338), where elephants selected sand forest and closed woodland over all other habitat types during in the dry season, and selected *Acacia* woodland in the wet season (Table S1). Similar to iSimangaliso, we did not observe differences in resource selection between day and night (Pillai's Trace = 2.0032, *F*_70, 126_ = 0.72, *P* = 0.9328), and resource use did not consistently correspond with the relative availability of habitats within the reserve (Figure 5). In Pilanesberg, resource selection differed between seasons (Pillai's Trace = 1.0712, *F*_36, 276_ = 1.67, *P* = 0.0128), but was consistent between day and night (Pillai's Trace = 1.0270, *F*_72, 276_ = 0.79, *P* = 0.8812), similar to iSimangaliso and Phinda. Elephants in Pilanesberg tended to select *Combretum*, *Faurea*, and *Acacia caffra* woodland over other habitat types during both the wet and dry seasons, but varied in their selection of grassland and mixed *Acacia* woodland among seasons (Table S1). Furthermore, in contrast to iSimangaliso and Phinda, resource selection more closely followed the relatively availability of habitats (Figure 5). Overall, despite the failure to observe temporal day vs. night differences in resource selection in iSimangaliso that would provide support for our temporal refuge hypothesis, the differences in resource selection patterns we observed among reserves provides further support for our spatial refuge hypothesis. In particular, selection of forest plantation and dry forest in favor of available habitat in iSimangaliso, regardless of season, suggests that restricted space use patterns are related to the avoidance of a particular area rather than to the availability of suitable habitat.

## Discussion

Our study suggests that chronic stress hormone concentrations are associated with restricted space use and altered habitat preferences that resemble a facultative refuge behavioral response. The elephant population in iSimangaliso displayed FGMs indicative of chronic stress and used a smaller portion of this reserve throughout the year. These results contrast with findings for other translocated populations with lower FGM concentrations, and other wild elephant populations [13], [29], [30], [31]. Restricted space use patterns indicative of refuge behavior have been documented for a variety of species, but few previous studies have linked the internal physiological status and selection of refugia [8], [32]. Our results suggest that if stressors are persistent and result in a chronic physiological state, populations will restrict space use and occupy refugia for an extended period of time.

Chronic stress response by elephants in iSimangaliso following translocation could be a consequence of delayed acclimatization. Previous studies on elephants have documented short-term elevations in FGM concentrations associated with poaching [33], hunting [34], fire [35], tourism [27], and translocation [24], [25]. Elephants selected for translocation to iSimangaliso exhibited baseline FGM concentrations prior to capture; however, FGM values did not return to baseline conditions within 30 days after the translocation event as found in previous studies of FGM responses to translocation of working elephants [24] and elephants allowed to navigate to their original home range [25]. One potential explanation is that it simply takes an extended period of time for wild elephants to acclimatize. For example, those elephant populations in our study with lower FGM concentrations were in reserves where initial translocations occurred 10–20 years prior to the initiation of the study, so it is possible that >6 years is required for physiological acclimatization following translocation to a new environment.

The spatial refuge behavioral response of elephants that we observed in iSimangaliso is potentially a consequence of avoiding the area associated with translocation and release. Because previous behavioral research has shown that there are sometimes long-term sociological and behavioral effects of traumatic events on elephants [36], [37], a persistent stress response could be attributed to the experience of a population or family group with the process of capture and translocation. Elevated stress responses to translocation have been reported with subsequent dispersal away from the release site for multiple species [4], including elephants [24]. Similarly, upon translocation to iSimangaliso, elephants were released in the Eastern Shores section, but quickly dispersed to the Western Shores section. All three separately introduced elephant family groups have subsequently resided in the latter section for 6 years post-release. This suggests that, given elephants were released on the Eastern Shores section, they could be avoiding the location associated with a translocation, a highly stressful event [4]. This avoidance following dispersal might be compounded by the presence of Lake St. Lucia, which could act as a barrier to movement between the two sections. However, during our study elephant family groups easily traversed the lake, crossing it 20 times to visit the Eastern Shores section for short periods (typically 24–48 hours) before returning to the Western Shores section.

The timing and frequency of human disturbances within iSimangaliso also could influence the refuge behavior pattern we observed. Wild elephant populations, similar to most wildlife, avoid areas associated with persistent interactions with humans [17]. The Eastern Shores section of iSimangaliso is open to the public and receives a consistently high level of tourism visitation, a factor known to elicit a physiological response in elephants [27]. By contrast, the Western Shores section is closed to the public, yet contains forest plantations that occasionally have a high amount of human disturbance by plantation workers, but which is localized to a particular stand. It is likely that elephants make trade-offs between relative risks associated with human disturbances within their environment. That is, elephants in iSimangaliso might utilize forest plantations, which are only intermittently visited by humans, and which occur in a matrix of native forest habitat that provides opportunities for the animals to escape disturbance, in favor of the Eastern Shores section, which more consistently receives human disturbance.

In addition to human disturbance, restricted foraging by elephants in iSimangaliso in dry forests and tree plantations could have influenced chronic FGM levels. Elevated elephant FGM concentrations might be related to nutritional stress and overall diet quality, where FGM concentrations are inversely related to the amount of nitrogen present in their diet [38]. The restricted space use patterns we observed in iSimangaliso, where elephants selected for and likely primarily consumed browse in dry forest and non-native tree plantations, likely further limited access to high quality forage regardless of season, and might have contributed to elevated FGM levels in that reserve. Therefore, in addition to potential human disturbance stressors, the impact of nutritional stress on chronic FGM concentrations is an area in need of research in translocated elephant populations.

The differences we observed in elephant space use patterns among populations did not correspond with our current understanding of how reserve shape, competition, and resource selection could restrict elephant space use. Elephants within fenced reserves have been shown to avoid areas in proximity to boundary fences [14], thus the shape of the reserves could influence elephant movement and space use. However, elephants in the most elongated and narrowest reserve (Phinda) utilized the highest portion (78%) of their reserve. By comparison, elephants in the round reserve (Pilanesberg), where we would expect less of an edge effect, utilized 55% of their reserve. African elephant family groups establish age- or size-related dominance hierarchies [39], which could result in competition between elephants that restricts space use patterns when populations are highly abundant and/or resources are limited [40]. While individual family groups could have exhibited greater competition and avoidance in iSimangaliso than the other two reserves, in general we would expect greater competition in reserves with higher elephant density [40]. In contrast, the reserve that exhibited the most restricted space use patterns contained the lowest elephant density (iSimangaliso, 0.04 elephants per km^2^) compared to the other two reserves (Phinda, 0.51 elephants per km^2^; Pilanesberg, 0.32 elephants per km^2^). The distribution of dry native forest and tree plantations within iSimangaliso, which were primarily limited to the Western Shores where we observed restricted spaces use patterns, suggests that these habitats could be limiting and selected over grasslands habitats that dominated the Eastern Shores. However, elephants generally are considered habitat generalists [41], [42]. Further, given that grasslands likely contained higher quality forage most similar to the donor site (Kruger National Park; [43]), particularly during the wet season [44], we feel selection of tree plantations and dry forests of the Western Shores is more likely due to elephants avoiding open areas (i.e. grasslands) and sources of human disturbance than nutritional attributes that typically drive habitat-related patterns in elephant movement [44].

The differences we observed in space use overlap between day and night in iSimangaliso suggest that elephants might slowly be adapting temporal refuge behavior in response to human disturbance. Despite restricting their use of space to the Western Shores, elephants in iSimangaliso continue to exhibit a state of chronic physiological stress. If utilizing forest plantation is a persistent stressor to elephants in iSimangaliso, under the concept of allostasis an individual or population should continue to adapt and change its behavior to minimize the likelihood of encountering stressors [2]. However, elephants tend to be slow in developing novel movement patterns in response to changes in their environment [15]. Therefore, given variation in the timing and location of disturbances in forest plantations, developing reliable movement patterns is likely difficult and elephants might only slowly be adapting to stressors in the Western Shores. It also is possible that the elephants have finer-scale refuge behavior that we were unable to detect at the scale of our analysis, such as avoidance of roads during periods of peak use by plantation workers. Future research is needed to evaluate if elephants in iSimangaliso continue to maintain an elevated physiological state, and if they modify their fine-scale spatial and temporal behavior over time.

Chronic stress and elephant refuge behavior could have a number of potential short and long-term consequences to elephant health, human safety, tourism, ecosystem processes, and biodiversity. Two months following the initial release of elephants in iSimangaliso, mortality of an 8 month-old male calf occurred, likely as a result of stressors associated with translocation and long, continuous movement of the family group post-release. This incident suggests that chronic stress is likely to be a problem for young animals, and that providing refugia to limit continuous movements could reduce the risk of future mortalities. The history of human deaths caused by elephants in the reserves included in this study, while anecdotal, suggests that chronic stress and refuge behavior might be linked to incidences of elephant aggression toward humans. In iSimangaliso, despite closure of the Western Shores to the public, elephants in a single family group have killed two reserve workers. Also, in Phinda, within 3–5 years after introduction, a female elephant killed a human. In Pilanesberg, by contrast, although there have been a number of elephant attacks on humans and one person has been killed, all attacks were by male elephants of which most if not all were in musth. Given that it is comparatively less common for female elephants to be aggressive [45], and stress associated with socially disruptive events like translocation have previously been associated with incidences of elephant aggression [37], [45], our findings collectively suggest that chronic stress and refuge behavior following translocation are at least loosely linked to elephant aggression toward humans. Refuge behavior by elephants also limits their tourism value. Elephants frequently are seen by tourists in Pilanesberg and Phinda, but rarely are seen in iSimangaliso, where the opportunity for viewing elephants was one of the primary reasons for their reintroduction. Finally, the repeated use of refugia by elephants over an extended period of time could lead to extensive habitat modification [46] and potentially to loss of biodiversity [47]. In the case of iSimangaliso, refuge behavior also could exacerbate the damage to commercially valuable trees in forest plantations.

Accounting for refuge behavior has important implications to our understanding of elephant space use. Seasonal variation in the spatial distribution of resources, primarily forage and water availability [13], as well as social interactions [48] and the shape of fenced reserves [14], are known to be key drivers of elephant space use. In addition to these factors, physiological state could influence space use and resource selection patterns. For example, in Pilanesberg and Phinda, elephants generally used resources in proportion to their availability (Figure 5). In contrast, elephants in iSimangaliso exhibited restricted spaces use patterns and selected forest plantations on the Western Shores in favor of native habitats. This does not rule out the possibility that elephants in Pilanesberg and Phinda exhibited refuge behavior over shorter periods of time or that they identified areas as refugia. It is likely that elephants in Phinda and Pilanesberg identified refugia that allowed them to recover following exposure to a stressor. For example, Woolley et al. [35] documented that, following a catastrophic fire event in Pilanesberg, elephants exhibited a short-term elevation in stress hormone levels and moved to the northern portion of the reserve, which is designated as a “wilderness zone” closed to tourists. Thus, the availability of refugia, when needed, is likely critical to successfully avoiding development of a chronic physiological state.

The identification of refugia is particularly important in South Africa, where elephants are increasingly restricted to fenced reserves [49]. The use of fences in South Africa generally has been effective at limiting elephant movements and potential human-elephant conflict [50], [51]. However, our findings suggest that issues of human-elephant conflict and refuge behavior within fenced reserves need to be addressed. One potential solution to this problem is to identify areas that can serve as refugia for elephants (such as wilderness zones as in Pilanesberg) and limit human disturbance in those areas. Alternatively, managers might identify specific areas and periods when refugia are needed, similar to the current concept of virtual fences used to mitigate human-elephant conflict [51]. For example, in iSimangaliso, where an individual female in each family group is monitored with a Global Positioning System collar linked to a cellular phone network, managers are using real-time elephant movement data in combination with computer technology based on geospatial maps, to send a notification message to one or more cell phones any time a collared elephant moves into a pre-determined zone, such as across a reserve border [51]. Similarly, if reserve managers are able to identify refugia spatially or predict via movement patterns when elephants are exhibiting refuge behavior, they could limit human disturbance to that area for a period of time, and potentially provide corridors into or among refugia to mitigate the risk of chronic stress and potentially dangerous human-elephant interactions.

In summary, managers considering the translocation or reintroduction of wildlife should consider the possibility of chronic stress and potential consequences of refuge behavior. Chronic stress is common following wildlife translocation, and has been associated with reproductive failure, increased predation risk, disease risk, and movement away from the release site [4], [52]. Our results suggest that chronic stress is associated with refuge behavior in translocated elephants, and we predict that it is likely to occur as a common facultative response in other species following translocation. Thus, future efforts to predict when, where, and to what extent wildlife populations will exhibit refuge behavior could likely be improved by an understanding of their physiological response.

## Materials and Methods

### Ethics Statement

The collection of elephant fecal samples and field observation techniques were approved by the Animal Ethics Sub-committee of the University of KwaZulu-Natal Ethics Committee (permit reference 012/09/Animal).

### Study Areas

We selected three reintroduced elephant populations in South Africa, which were each contained by electrified boundary fences: Pilanesberg Game Reserve (25°8′–25°22′S, 26°57′–27°13′E), iSimangaliso Wetland Park (28°49′–27°55′S, 32°68′–32°22′E), and Phinda Private Game Reserve (27°92′–27°68′S, 32°44′–32°20′E). Most individual elephants within our three study sites were translocated from Kruger National Park, or were the offspring of such animals [53]. Exceptions were 10 individuals at Phinda Private Game Reserve brought in 1993 from Gonarhezou in Zimbabwe [23], and six individuals (two from US captive populations, two from Namibia, and two from Mabula Game Reserve) that were released in Pilanesberg National Park [54].

Pilanesberg National Park (hereafter referred to as Pilanesberg), located in the North West Province, is 560 km^2^ in size and is composed of hilly terrain containing a mix of open grasslands and closed *Acacia* and broad-leaf bushveld [29]. We classified habitats based on seven major vegetation types in the park [14], [55]: (1) *Acacia caffra* woodland, (2) *A. karoo* woodland, (3) *A. mellifera* woodland, (4) *Combretum* woodland, (5) *Faurea* woodland, (6) mixed *Acacia* woodland, and (7) grassland. Fifty-eight male and 37 female elephants were reintroduced from 1981 to 1998, primarily from Kruger National Park [56]. In 2004 there were at least 16 family groups [57], and by 2009 there were approximately 180 individual elephants in the park (S. Dell, Pilanesberg National Park, personal communication).

Phinda Private Game Reserve (hereafter referred to as Phinda), located in the KwaZulu-Natal Province, is 180 km^2^ in size and contains a range of habitats that include sweet lowveld bushveld, Natal low bushveld, and coastal bushveld [58]. We used existing land use and vegetation maps created by Noel van Rooyen and Simon Morgan for reserve management to classify habitats into eight categories: (1) *Acacia* woodland, (2) human habitation, (3) open grassland, (4) closed woodland, (5) riverine and wetland, (6) sand forest, (7) Lebombo thicket, and (8) palmveld. Managers released 54 orphan elephants in 1992–1994 and 3 mature adult bulls in 2003 [15]. In 2009, there are at least five family groups, and the total population in 2010 was estimated to be 93 individuals (T. Burke, Phinda Private Game Reserve, personal communication).

The iSimangaliso Wetland Park is located on the eastern coast in KwaZulu-Natal Province. It is 602 km^2^ in size and is composed of the Eastern Shores section (273 km^2^) bordered by fencing to the north and south, by the Indian Ocean to the east and the estuary of Lake St. Lucia to the west; and the Western Shores section (329 km^2^) bordered by Lake St. Lucia to the east and electrified fence along its other boundaries. We used existing vegetation and land use maps created by Noel van Rooyen for park management to classify iSimangaliso into eight major habitat types: (1) tree plantations (composed of either *Eucalyptus globulus* or *Casuarina equisetifolia*), (2) dry forest, (3) lowland forest, (4) marsh and swamp, (5) freshwater lake, (6) grassland, (7) human settlement, and (8) open beach. We did not consider the estuarine Lake St. Lucia as available habitat in our analysis. The reintroduction of elephants to iSimangaliso was initiated in 2001 with the translocation of a 24 elephants (15 females and 9 males) from Hluhluwe-iMfolozi Park (originally from Kruger National Park), and in 2002 and 2003 with two additional family groups directly from Kruger National Park.

### Stress hormone data

From 2000 to 2006, we sampled FGM concentrations of elephants in each of the three reserves. In the field, fecal samples were collected opportunistically by trained employees of the reserves or by the University of KwaZulu-Natal. On average, samples from Phinda, Pilanesberg, and iSimangaliso were collected within 30 min, 10 hrs, and 20 hrs respectively. Across all reserves, time between deposition and collection for all samples used was <72 hrs, and similar to other FGM-based studies on elephant [25], [34], [59]. We recorded the approximate age of the sample as well as the location of collection, but were unable to consistently identify which individual or family group deposited the sample. Samples for laboratory analysis were collected by opening, and taking a portion from the center of the bolus [25], [34]. After collection, samples were immediately treated with a 2% acetic acid solution and frozen for shipment [60]. In the laboratory, samples were stored at −80°C, freeze-dried, ground, and sifted through a stainless steel mesh. We extracted FGM from the feces using corticosterone I^125^ radioimmunoassay kits (MP Biomedicals, Costa Mesa, CA) following validated and established protocols [28]. Inter-assay variation for 11 assays was 7.3% and average intra-assay variation was 3.9%.

We conducted a nested analysis of variance (ANOVA) to determine if significant differences occurred in mean FGM concentrations of elephants among the three reserves, and if differences within reserves occurred between years. In addition, we evaluated if FGM concentrations followed a pattern of variation between seasons (wet and dry) similar to that seen in previous studies [61], [38]. We partitioned data into annual wet and dry seasons based on rainfall patterns for our study areas, where the wet season occurred from November to April, and the dry season occurred from May to October [30], [54].

### Location Data

From 2004 to 2007, GPS collars were placed on a single adult female individual in each of 14 family groups (iSimangaliso n = 3, Phinda n = 5, Pilanesberg n = 6). Because adult female elephants live in cohesive family units, we assumed that GPS collars deployed on adult female elephants capture the movements of an entire family group [17]. All collars were programmed to record elephant locations at predetermined intervals (ranging from 30 min to 12 hrs depending on the individual elephant) and to transmit coordinates by Global System for Mobile Communications (GSM) cell phone signal or satellites to a ground station where they were stored on a master computer [15]. We omitted locations in Pilanesberg from September 2005 to September 2006 due to a catastrophic fire that altered elephant space use patterns [35]. We also omitted locations in Phinda prior to September 2005 due to removal of a section of fence at that time that allowed for expansion of the reserve [15]. While locational data were not validated, location error was relatively low (<50 m) based on evaluations of similar GPS collars on elephants [13], [14].

### Analysis of Space Use Patterns

In analyzing elephant space use, we first wanted to identify distinct, biologically meaningful time intervals among which we could compare space use patterns by family groups over time. Elephant space use patterns consistently vary between two annual seasons based on rainfall (i.e. wet and dry seasons) [13], [29]. Given that elephants at the donor site (Kruger National Park) also exhibit distinctive wet and dry season movement patterns [62], we predicted that translocated elephants at our study sites would similarly exhibit seasonal movement patterns [30], [31].

We developed seasonal utilization distributions (UDs) [63] to estimate space use for each season during which an elephant continuously wore a GPS collar. Between 2004 and 2007, within each season we captured ≥300 locations (*x* ¯ = 303.51, SE = 8.33, range = 90–370) of elephants separated by 12±2 hours in each of our three study sites. We represented space use by each elephant family group during each season by creating 95% fixed kernel UDs using the plug-in method of bandwidth selection [64]. Because elephant space use is limited by hard boundaries (i.e. electric fences) at each reserve, we trimmed each UD by the reserve boundary and standardized the remaining UD value so that cell values in each UD summed to 1.0.

To evaluate the spatial refuge hypothesis, that elephant family groups with high FGM levels exhibit restricted space use, we compared the proportion of a reserve utilized by elephants among reserves. Because each reserve was completely fenced around the entire perimeter (except for portions of iSimangaliso bordered by lake or ocean) and fences created an edge effect influencing elephant movement [14], home range size was likely influenced by reserve size [30]. Therefore, because reserves were different sizes (180 km^2^ to 602 km^2^), we evaluated elephant space use based on the percent of the reserve occupied by the UD contour in addition to home range size estimates. We evaluated the home range size and proportion of the reserve utilized by each family group during each season for normality and compared among reserves, family groups, years, and seasons using a nested factorial ANOVA. In the ANOVA, reserve, year and season were fixed effects, elephant family group was nested within reserve, and home range size or proportion of the reserve utilized was the dependent variable.

To evaluate the temporal refuge hypothesis, that elephants with elevated FGM levels exhibit different behavioral patterns in day vs. night, we compared day home range size to night home range size within each reserve. Given that tourist game drive traffic and, in the case of iSimangaliso, forestry operations, primarily occur during daylight, we hypothesized that there might be differences in day and night space use by elephants. We categorized locations into day or night separately for each season and computed UDs for each family group using procedures described above. We defined day as between 0800 and 1900, and night as between 2100 and 0600. We omitted locations between 1900 and 2100 and 0600 and 0800 due to seasonal variations in the time of sunrise and sunset, and because some guided tourism viewing occurs during those periods. We computed home range sizes for both day and night within each season for each elephant, and evaluated if there were significant differences in home range size between day and night within each reserve individually using a factorial nested ANOVA. In the ANOVA, elephant family group was a fixed effect, season was nested within elephant family group, time (in terms of UDs based on daytime or nighttime locations) was nested within season and elephant family group, and home range size was the dependent variable.

To determine the extent to which elephants used the same area by day as by night, we evaluated space use overlap by individual family groups between day and night within each season using a volume of intersection (VI) analysis [65], [66]. The VI index measures overlap in space use between two UDs (as distinct from polygon overlap). Volume of intersection scores range from 0–1, where a VI score of 1 indicates perfect overlap of the UDs. Therefore, we interpreted higher VI scores as evidence of the repeated use of space between day and night. To account for potential day-night variation in highly utilized areas, we computed VI scores for both the home range scale of 95% fixed-kernel UDs, and core area scale of 50% fixed-kernel UDs [67]. We log-transformed VI scores and used a nested ANOVA to test the null hypothesis that no difference occurred in VI scores among reserves [68]. In the ANOVA, reserve, year and season were fixed effects, elephant family group was nested within reserve, and the VI score for comparing day vs. night space use was the sampling unit.

### Analysis of resource selection

We assessed resource selection by elephants in each reserve using a weighted compositional analysis [69]. We utilized the 95% fixed-kernel UD for each day and night period and summed UD values for each habitat type. We divided the summed UD values for each habitat type by total UD score to get weighted proportional use of each habitat type by an elephant. We substituted 0.5% for 0 for all non-used habitats [70] and subtracted log-transformed use data from log-transformed availability data (at the reserve level) for each elephant at each sampling interval to calculate the difference in log-ratios [69], [71]. We evaluated if overall selection occurred using Wilk's lambda statistic to test if the mean vector of log-ratio differences differed from a vector of zeros, and when selection occurred, we ranked habitats based on their relative utilization [71]. We tested for effects of season (wet vs. dry) and time of day (day vs. night) on log-ratio difference values for each habitat type in each reserve using a nested multivariate analysis of variance (MANOVA) [72]. In the MANOVA, elephant family group was the fixed effect, season was nested within elephant family group, time of day was nested within season and elephant family group, and the log-ratio differences were the sampling unit.

## Supporting Information

Table S1 Matrices and habitat rankings of African elephant seasonal resource selection in iSimangaliso Wetland Park (A), Phinda Private Game Reserve (B), and Pilanesberg National Park (C), South Africa. The + or − sign values within habitat comparisons indicate direction of selection based on positive or negative *t*-values; and +++ or −−− indicate both the direction of selection and if significant differences occurred at *P*<0.05. A rank of 1 indicates the highest level of selection.
(DOCX)
