# Supplementary material for: Physiological Stress and Refuge Behavior by African Elephants
Source: PLoS One. 2012 Feb 22;7(2):e31818. doi: 10.1371/journal.pone.0031818 (PMC3284500; doi:10.1371/journal.pone.0031818)
Supplement: Table S1 — Matrices and habitat rankings of African elephant seasonal resource selection in iSimangaliso Wetland Park (A), Phinda Private Game Reserve (B), and Pilanesberg National Park (C), South Africa. The + or − sign values within habitat comparisons indicate direction of selection based on positive or negative t-values; and +++ or −−− indicate both the direction of selection and if significant differences occurred at P<0.05. A rank of 1 indicates the highest level of selection. (DOCX) [file pone.0031818.s001.docx]

**Table S1.** Matrices and habitat rankings of African elephant seasonal resource selection in iSimangaliso Wetland Park (A), Phinda Private Game Reserve (B), and Pilanesberg National Park (C), South Africa. The + or ‒ sign values within habitat comparisons indicate direction of selection based on positive or negative *t*-values; and +++ or ‒ ‒ ‒ indicate both the direction of selection and if significant differences occurred at *P*<0.05. A rank of 1 indicates the highest level of selection.

(A)

| **iSimangaliso: dry season** | | |  |  |  |  |  |  |  |
| --- | --- | --- | --- | --- | --- | --- | --- | --- | --- |
|  | Plantation | Lowland forest | Dry forest | Grassland | Marsh and Swamp | Open beach | Freshwater lake | Human settlement | **Rank** |
| Plantation | . | +++ | + | +++ | +++ | +++ | +++ | +++ | 1 |
| Lowland forest | ‒ ‒ ‒ | . | + | + | +++ | +++ | +++ | +++ | 2 |
| Dry forest | ‒ | ‒ | . | + | +++ | +++ | +++ | +++ | 3 |
| Grassland | ‒ ‒ ‒ | ‒ | ‒ | . | +++ | +++ | +++ | +++ | 4 |
| Marsh and Swamp | ‒ ‒ ‒ | ‒ ‒ ‒ | ‒ ‒ ‒ | ‒ ‒ ‒ | . | + | + | +++ | 5 |
| Open beach | ‒ ‒ ‒ | ‒ ‒ ‒ | ‒ ‒ ‒ | ‒ ‒ ‒ | ‒ | . | + | + | 6 |
| Freshwater lake | ‒ ‒ ‒ | ‒ ‒ ‒ | ‒ ‒ ‒ | ‒ ‒ ‒ | ‒ | ‒ | . | + | 7 |
| Human settlement | ‒ ‒ ‒ | ‒ ‒ ‒ | ‒ ‒ ‒ | ‒ ‒ ‒ | ‒ ‒ ‒ | ‒ | ‒ | . | 8 |
|  |  |  |  |  |  |  |  |  |  |
| **iSimangaliso: wet season** | | |  |  |  |  |  |  |  |
| Plantation | . | +++ | + | +++ | +++ | +++ | +++ | +++ | 1 |
| Dry forest | ‒ | +++ | . | +++ | +++ | +++ | +++ | +++ | 2 |
| Lowland forest | ‒ ‒ ‒ | . | ‒ ‒ ‒ | + | + | +++ | + | +++ | 3 |
| Grassland | ‒ ‒ ‒ | ‒ | ‒ ‒ ‒ | . | + | +++ | +++ | +++ | 4 |
| Marsh and Swamp | ‒ ‒ ‒ | ‒ | ‒ ‒ ‒ | ‒ | . | +++ | + | +++ | 5 |
| Freshwater lake | ‒ ‒ ‒ | ‒ | ‒ ‒ ‒ | ‒ ‒ ‒ | ‒ | +++ | . | +++ | 6 |
| Open beach | ‒ ‒ ‒ | ‒ ‒ ‒ | ‒ ‒ ‒ | ‒ ‒ ‒ | ‒ ‒ ‒ | . | ‒ ‒ ‒ | +++ | 7 |
| Human settlement | ‒ ‒ ‒ | ‒ ‒ ‒ | ‒ ‒ ‒ | ‒ ‒ ‒ | ‒ ‒ ‒ | ‒ ‒ ‒ | ‒ ‒ ‒ | . | 8 |

(B)

| **Phinda: dry season** | | |  |  |  |  |  |  |  |
| --- | --- | --- | --- | --- | --- | --- | --- | --- | --- |
|  | Sand forest | Closed woodland | Palmveld | Riverine and wetland | Human habitation | Open grassland | *Acacia* woodland | *Lebombo* thicket | **Rank** |
| Sand forest | . | +++ | +++ | +++ | +++ | +++ | +++ | +++ | 1 |
| Closed woodland | ‒ ‒ ‒ | . | +++ | +++ | +++ | +++ | +++ | +++ | 2 |
| Palmveld | ‒ ‒ ‒ | ‒ ‒ ‒ | . | + | + | + | + | +++ | 3 |
| Riverine and wetland | ‒ ‒ ‒ | ‒ ‒ ‒ | ‒ | . | + | + | +++ | +++ | 4 |
| Human habitation | ‒ ‒ ‒ | ‒ ‒ ‒ | ‒ | ‒ | . | + | +++ | +++ | 5 |
| Open grassland | ‒ ‒ ‒ | ‒ ‒ ‒ | ‒ | ‒ | ‒ | . | + | +++ | 6 |
| *Acacia* woodland | ‒ ‒ ‒ | ‒ ‒ ‒ | ‒ | ‒ ‒ ‒ | ‒ ‒ ‒ | ‒ | . | +++ | 7 |
| *Lebombo* thicket | ‒ ‒ ‒ | ‒ ‒ ‒ | ‒ ‒ ‒ | ‒ ‒ ‒ | ‒ ‒ ‒ | ‒ ‒ ‒ | ‒ ‒ ‒ | . | 8 |
|  |  |  |  |  |  |  |  |  |  |
| **Phinda: wet season** | | |  |  |  |  |  |  |  |
| *Acacia* woodland | + | +++ | + | + | +++ | +++ | . | +++ | 1 |
| Closed woodland | + | . | +++ | ‒ ‒ ‒ | ‒ | +++ | ‒ ‒ ‒ | + | 2 |
| Riverine and wetland | + | +++ | +++ | . | + | + | ‒ | +++ | 3 |
| Sand forest | . | ‒ | +++ | ‒ | ‒ | + | ‒ | + | 4 |
| *Lebombo* thicket | ‒ | ‒ | + | ‒ ‒ ‒ | ‒ ‒ ‒ | + | ‒ ‒ ‒ | . | 5 |
| Palmveld | ‒ ‒ ‒ | ‒ ‒ ‒ | . | ‒ ‒ ‒ | ‒ ‒ ‒ | ‒ | ‒ ‒ ‒ | ‒ | 6 |
| Open grassland | ‒ | --- | + | ‒ ‒ ‒ | ‒ ‒ ‒ | . | ‒ ‒ ‒ | ‒ | 7 |
| Human habitation | + | + | +++ | ‒ | . | +++ | ‒ | +++ | 8 |

(C)

| **Pilanesberg: dry season** | | |  |  |  |  |  |  |
| --- | --- | --- | --- | --- | --- | --- | --- | --- |
|  | *Combretum* woodland | *Faurea* woodland | *Acacia caffra* woodland | Mixed *Acacia* woodland | *Acacia karoo* woodland | Grassland | *Acacia melifera* woodland | **Rank** |
| *Combretum* woodland | . | + | + | +++ | +++ | +++ | +++ | 1 |
| *Faurea* woodland | ‒ | . | + | + | + | +++ | +++ | 2 |
| *Acacia caffra* woodland | ‒ | ‒ | . | + | +++ | +++ | +++ | 3 |
| Mixed *Acacia* woodland | ‒ ‒ ‒ | ‒ | ‒ | . | + | +++ | +++ | 4 |
| *Acacia karoo* woodland | ‒ ‒ ‒ | ‒ | ‒ ‒ ‒ | ‒ | . | +++ | +++ | 5 |
| Grassland | ‒ ‒ ‒ | ‒ ‒ ‒ | ‒ ‒ ‒ | ‒ ‒ ‒ | ‒ ‒ ‒ | . | +++ | 6 |
| *Acacia melifera* woodland | ‒ ‒ ‒ | ‒ ‒ ‒ | ‒ ‒ ‒ | ‒ ‒ ‒ | ‒ ‒ ‒ | ‒ ‒ ‒ | . | 7 |
|  |  |  |  |  |  |  |  |  |
| **Pilanesberg: wet season** | |  |  |  |  |  |  |  |
| *Combretum* woodland | . | + | +++ | +++ | +++ | + | +++ | 1 |
| *Faurea* woodland | ‒ | . | + | +++ | +++ | + | +++ | 2 |
| *Acacia caffra* woodland | ‒ ‒ ‒ | ‒ | . | +++ | +++ | + | +++ | 3 |
| Grassland | ‒ | ‒ | ‒ | + | + | . | +++ | 4 |
| *Acacia karoo* woodland | ‒ ‒ ‒ | ‒ ‒ ‒ | ‒ ‒ ‒ | + | . | ‒ | + | 5 |
| Mixed *Acacia* woodland | ‒ ‒ ‒ | ‒ ‒ ‒ | ‒ ‒ ‒ | . | ‒ | ‒ | + | 6 |
| *Acacia melifera* woodland | ‒ ‒ ‒ | ‒ ‒ ‒ | ‒ ‒ ‒ | ‒ | ‒ | ‒ ‒ ‒ | . | 7 |
